# Iguratimod alleviates tubulo-interstitial injury in mice with lupus

**DOI:** 10.1080/0886022X.2022.2058962

**Published:** 2022-04-06

**Authors:** Leixi Xue, Jiajun Xu, Wentian Lu, Jinxiang Fu, Zhichun Liu

**Affiliations:** a Department of Rheumatology and Immunology, The Second Affiliated Hospital of Soochow University, Suzhou, China; b Department of Hematology, The Second Affiliated Hospital of Soochow University, Suzhou, China

**Keywords:** Iguratimod, lupus nephritis, renal interstitial fibrosis, epithelial-to-mesenchymal transition

## Abstract

**Introduction:**

Tubulo-interstitial injury is a poor prognostic factor for lupus nephritis (LN). Here, we tested whether iguratimod could inhibit tubulo-interstitial injury in LN.

**Methods:**

MRL/lpr mice, an animal model of lupus, were treated with iguratimod or vehicle solution. Pathological changes of kidney were evaluated blindly by the same pathologist. Renal type I collagen (COL-I), IgG, E-cadherin, fibroblast-specific protein 1 (FSP-1) were detected by immunofluorescence, immunohistochemical staining or quantitative real-time PCR. After treated with transforming growth factor β1 (TGF-β1) and iguratimod, E-cadherin, fibronectin, Smad2/3, p38 MAPK, p-Smad2/3, and p-p38 MAPK, β-catenin and TGF-β type II receptor (TGFβRII) in HK2 cells were measured by western blotting, quantitative real-time PCR or immunofluorescence.

**Results:**

Iguratimod reduced immune deposition along the tubular basement membrane, inhibited the tubulo-interstitial infiltration of inflammatory cells, and alleviated tubular injury in MRL/lpr mice. Moreover, Iguratimod eased the tubulo-interstitial deposition of collagen fibers, which was confirmed by decreased expression of COL-I. Furthermore, iguratimod suppressed the expression of FSP-1 and increased that of E-cadherin in renal tubular epithelial cells. In HK2 cells cultured with TGF-β1, iguratimod treatment not only reversed cellular morphological changes, but also prevented E-cadherin downregulation and fibronectin upregulation. In addition, iguratimod inhibited phosphorylation of TGFβRII, Smad2/3 and p38 MAPK in HK2 cells treated with TGF-β1, and also blocked nuclear translocation of β-catenin.

**Conclusion:**

Iguratimod eased tubulo-interstitial lesions in LN, especially tubulo-interstitial fibrosis, and might have potential as a drug for inhibiting the progression of tubulo-interstitial fibrosis in LN.

## Introduction

Lupus nephritis (LN) is one major manifestation of systemic lupus erythematosus (SLE), a common autoimmune disease that produces many types of autoantibodies and involves multiple organs, and is an important cause of secondary glomerulonephritis [1]. Glomerulus is generally the initial site of renal damage in LN. Glomerulonephritis is closely related to the disease activity of patients with SLE, and is often the focus of clinical treatment [2]. However, studies have confirmed that compared with glomerular lesions, tubulo-interstitial damage often portends poor long-term renal prognosis [3]. So far, tubulo-interstitial injury in LN has received relatively less attention.

Iguratimod is a new antirheumatic drug that suppresses the production of tumor necrosis factor α (TNFα), interleukin 6 (IL-6), and IL-8 in lipopolysaccharide-stimulated THP-1 cells [4], and inhibits cartilage and bone damage in DBA/1J mice with type II collagen (COL-II)-induced arthritis [5]. The Asia Pacific League of Associations for Rheumatology has recommended iguratimod to treat rheumatoid arthritis (RA) [6]. Iguratimod might also have therapeutic effects against other diseases. Iguratimod ameliorates acute and chronic experimental autoimmune encephalomyelitis by inhibiting inflammatory cell infiltration and immune cell activation, partly through suppressing the NF-κB signaling pathway, supporting its therapeutic potential for acute and chronic multiple sclerosis [7]. It also plays a protective role in mice with dextran sulfate sodium-induced colitis by regulating Th17/Treg cells and anti-inflammatory effects, suggesting that iguratimod could serve as a novel therapeutic drug for treating inflammatory bowel disease [8].

Lupus mice administered with iguratimod have less proteinuria, lower glomerular injury and lower vasculitis scores than vehicle-treated controls [9]. However, whether iguratimod could alleviate tubulo-interstitial injury in LN remains unclear. Thus, we aimed to determine the therapeutic effects of iguratimod on tubulo-interstitial injury.

## Materials and methods

### Mouse models

MRL/lpr and MRL/MpJ mice (Shanghai Slack Laboratory Animal Co. Ltd., Shanghai, China) were bred and maintained in the animal facility at the Second Affiliated Hospital of Soochow University. The Ethics Committee at Soochow University approved the animal study protocols (Approval No. SUDA20200225A10), Suzhou, China. The study was conducted in accordance with the National Institutes of Health guide for the care and use of Laboratory animals (NIH Publications No. 8023, revised 1978). Iguratimod (Simcere Pharmaceutical, Nanjing, China) was dissolved in 1% carboxymethyl cellulose (CMC) to a concentration of 1.5 mg/mL. When the MRL/lpr mice reached the age of 8 weeks, they were given 1.5 mg/mL iguratimod (0.02 mL/mg/d) or 1% CMC (0.02 mL/mg/d) by gavage then sacrificed 16 weeks later.

### Serum analysis

Serum creatinine and blood urea nitrogen levels were determined using an automated biochemical analyzer (Rayto, Shenzhen, China).

### Cell culture

Human proximal tubular epithelial cells (HK2 cells) from the Cell Bank of the Chinese Academy of Sciences were cultured in a 5% CO_2_ atmosphere at 37 °C in Gibco DMEM/F-12 medium supplemented with 10% fetal bovine serum (both from Thermo-Fisher Scientific Inc., Waltham, MA, USA), 100 U/mL penicillin G sodium, and 100 μg/mL streptomycin sulfate (Beyotime, China). The cells were seeded at a density of 70% and incubated with TGF-β1 (PeproTech Inc., USA) at the indicated concentrations and times, or incubated for 2 h with iguratimod followed by TGF-β1.

### Quantitative real-time (qRT) PCR

Total RNA was extracted from kidney tissues or HK2 cells using TRIzol (Invitrogen, Carlsbad, CA, USA) as described by the manufacturer. Quantitative real-time PCR of triplicate samples proceeded using SsoFast™ EvaGreen Supermix and the CFX96 Real-Time PCR Detection System (both from Bio-Rad Laboratories Inc., Hercules, CA, USA), with the following respective forward and reverse primers (5′ → 3′): mouse glyceraldehyde-3-phosphate dehydrogenase (GAPDH), CCT CGT CCC GTA GAC AAA ATG and TGA GGT CAA TGA AGG GGT CGT (133 bp); mouse COL-I, GAG AGG TGA ACA AGG TCC CG and AAA CCT CTC TCG CCT CTT GC (153 bp); mouse fibroblast-specific protein 1 (FSP-1), GTG TCC ACC TTC CAC AAA TAC TCA and AAC TTC ATT GTC CCT GTT GCT G (174 bp); mouse E-cadherin, CGA CCG AAG TGA CTC GAA AT and TCA GAA CCA CTG CCC TCG TAA T (188 bp); human GAPDH, CTT TGG TAT CGT GGA AGG ACT C and GTA GAG GCA GGG ATG ATG TTC T (132 bp); human E-cadherin, AGC CCC GCC TTA TGA TTC TC and TTG CCC CAT TCG TTC AAG TAG (132 bp); human fibronectin, CAG CAA GCA AAG GTC GG and CAA AGC CTA AGC ACT GGC ACA A (184 bp). The endogenous control to normalize for differences in the amount of total RNA in each sample was GAPDH. Relative changes in gene expression were calculated as fold differences using the 2^−ΔΔCt^ method.

### Western blotting

Total proteins were extracted from HK2 cells with RIPA Lysis Buffer (Beyotime, China), and nuclear and cytoplasmic proteins were extracted using NE-PER Nuclear and Cytoplasmic Extraction Reagents (Thermo-Fisher Scientific Inc., USA). The proteins were then separated by SDS-PAGE and electrophoretically transferred onto polyvinylidene fluoride membranes that were incubated overnight at 4 °C with rabbit polyclonal antibodies specific for GAPDH (1:2500) and fibronectin (1:5000) (Abcam, UK), or rabbit monoclonal antibodies specific for E-cadherin (1:20,000) (Abcam, UK), β-catenin (1:5000) (Abcam, UK), TGF-β type II receptor (TGFβRII) (1:2000) (Abcam, UK), p-TGF-β type II receptor (TGFβRII) (1:5000) (Abcam, UK), Smad2/3 (1:1000) (Cell Signaling Technology, USA), p-Smad2/3 (1:1000) (Cell Signaling Technology, USA), p38 MAPK (1:1000) (Cell Signaling Technology, USA), and p-p38 MAPK (1:1000) (Cell Signaling Technology, USA), or mouse monoclonal antibodies specific for histone H3 (1:1000) (Beyotime, China). Proteins were detected using a LumiGLO chemiluminescent substrate system (Cell Signaling Technology, USA).

### Renal histopathology

The kidneys were dissected, fixed in 4% paraformaldehyde, embedded in paraffin, and stained with hematoxylin and eosin, Masson trichrome, and Sirius Red. The sections were evaluated blindly by one pathologist, as described [10–12]. Tubular disease (atrophy, dilation, casts) and interstitial inflammation of the renal cortex were respectively scored from 0 to 4 as absent, or present in <25%, 25–50%, 50–75%, and >75% of one section). Interstitial fibrosis was graded on a scale of 0–3 as <5%, 5–10%, 11–25%, and >25%, respectively.

### Immunohistochemical staining

Kidney sections were immunohistochemically stained using an established protocol [13]. Deposits of IgG were detected using horseradish peroxidase-conjugated goat anti-mouse IgG (Servicebio, China), followed by DAB-chromogen (Servicebio, China). Sections were then incubated with rabbit anti-mouse polyclonal antibodies (Servicebio, China) against Ki-67 and against caspase-3, followed by horseradish peroxidase-conjugated goat anti-rabbit IgG (Servicebio, China). Staining intensity was scored by a renal pathologist who was blinded to the groups of mice as described [12]. Ki-67(+) cells were scored according to their numbers/40× field, and caspase-3(+) cells were scored based on the absence of significant staining or occasional positive lymphocytes (0), rare tubular cell staining (1), frequent tubular cell staining (2).

### Immunofluorescence staining

Cells were indirectly stained with immunofluorescence according to established procedures. HK-2 cells grown on cover slips were washed three times with cold PBS and fixed in Immunol Staining Fix Solution (Beyotime, China) for 10 min. The cells were then extensively washed three times for 5 min each with Immunol Staining Scrubbing Solution (Beyotime, China). Nonspecific protein binding was blocked in Immunol Staining Blocking Buffer (Beyotime, China), then the cells were incubated at 4 °C overnight with the indicated primary antibody, followed by Alexa Fluor^®^ 488-conjugated goat antibody (Abcam, UK) for 60 min at 37 °C. Finally, the cells were stained with DAPI Staining Solution (Beyotime, China) to visualize nuclei and assessed by confocal laser scanning microscopy.

Kidney sections were incubated with specific primary antibodies against COL-I, FSP-1, and E-cadherin, followed by staining with Cy3-conjugated secondary antibodies (Servicebio, China). Immunoglobulin G deposits along the tubular basement membrane were detected using Alexa Fluor^®^ 488-conjugated goat anti-mouse IgG (Servicebio, China). The sections were then stained with DAPI and visualized by confocal laser scanning microscopy (Zeiss, Germany).

### Statistical analysis

Data were statistically analyzed using GraphPad Prism 5 (GraphPad Software Inc., San Diego, CA, USA) and are expressed as means ± SEM. Groups were compared using one-way ANOVA followed by Bonferroni multiple comparison tests. Estimates of renal damage were compared using non-parametric Kruskal-Wallis tests. All experiments *in vitro* were repeated at least four times to ensure reproducibility. Differences were considered significant at *p* < .05.

## Results

### Iguratimod reduced immune deposition along the tubular basement membrane and inflammatory cell infiltration of renal interstitium

**Figure 1.**
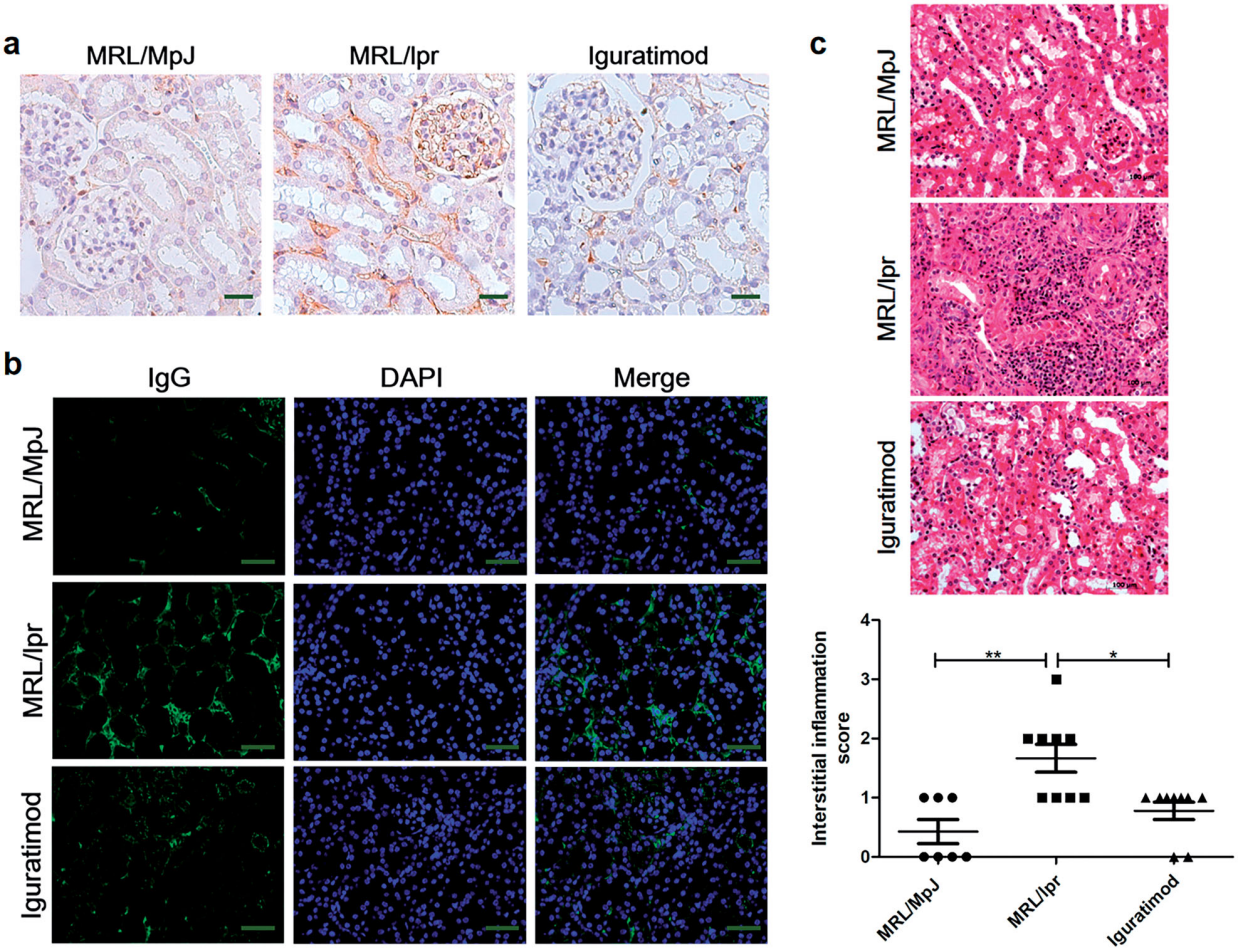
Iguratimod reduced IgG deposition along tubular basement membrane and inflammatory cell infiltration of renal interstitium. (a) IgG deposition in kidney detected by immunohistochemical staining (scale bar, 25 μm) and (b) immunofluorescence (scale bar, 100 μm), respectively. (c) Hematoxylin and eosin staining of renal interstitium shows infiltrative inflammatory cells (scale bar, 100 μm), and blind evaluation was performed. Results are shows as means ± SEM. *n* = 7, 9, 9, respectively. *p < .05, **p < .01, ***p < .001.

SLE is characterized by the production of a large number of autoantibodies, especially anti-dsDNA antibodies, which are pivotal in the pathogenesis of LN *via* deposition in the mesangial, subendothelial, subepithelial, or tubulo-interstitial regions [14]. As a result, immune deposition along the tubular basement membrane is quite prevalent in LN and is might be a potential trigger of the interstitial infiltration of inflammatory cells, tubular injury, and tubulo-interstitial fibrosis [15]. Therefore, we assessed immune deposition along the tubular basement membrane using immunohistochemical and immunofluorescent staining. A very small amount of IgG was deposited along the tubular basement membrane in MRL/MpJ mice, whereas the staining intensity was increased in MRL/lpr mice (Figure 1(a,b)). However, iguratimod significantly reduced immune deposition along the tubular basement membrane in MRL/lpr mice (Figure 1(a,b)).

We stained renal interstitial cells with hematoxylin and eosin to determine the effects of iguratimod on the infiltrative inflammatory cells. Figure 1(c) shows abundant infiltrative inflammatory cells around the renal tubules of MRL/lpr mice, whereas iguratimod significantly decreased the numbers of these cells.

### Iguratimod alleviated renal tubule injury

**Figure 2.**
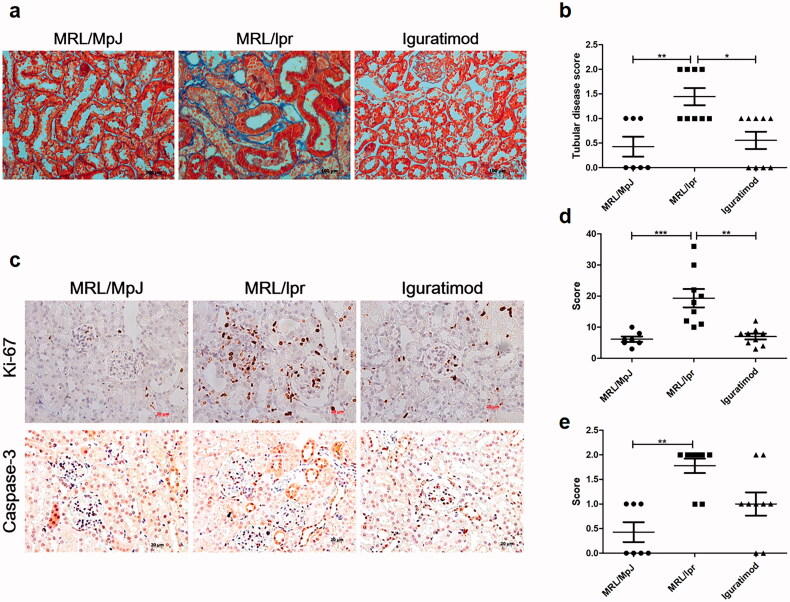
Iguratimod alleviated renal tubule injury. (a) Tubular injury was measured with Masson trichrome staining (scale bar, 100 μm). (b) Tubular disease (atrophy, dilation, casts) was evaluated blindly. (c) Ki-67 and caspase-3 were detected by immunohistochemical staining (scale bar, 20 μm). Scores for Ki-67(+) (d) and caspase-3(+) (e) cells. Data are shown as means ± SEM. *n* = 7, 9, 9, respectively. *p < .05, **p < .01, ***p < .001.

Figure 2(a,b) shows that iguratimod obviously decreased tubular atrophy, dilation, and casts in lupus mice (*p* < .01). Because tubular damage is closely associated with the abnormal proliferation and apoptosis of tubular epithelial cells [14,16], Ki-67 indicating cell proliferation and caspase-3 indicating apoptosis were evaluated using immunohistochemical staining [12,17]. Rare tubular epithelial cells were labeled by Ki-67 in MRL/MpJ mice, but a number of Ki-67(+) cells were found in the tubular area of MRL/lpr mice (Figure 2(c,d)). In parallel, iguratimod considerably decreased the number of Ki-67(+) cells in the tubular area of MRL/lpr mice (Figure 2(c,d)). The results of caspase-3 expression evaluation indicating apoptosis were consistent with those of proliferation; iguratimod also eased the apoptosis of tubular epithelial cells, but the difference did not reach statistical significance (Figure 2(c,e)).

### Iguratimod inhibited renal interstitial deposition of collagen fibers

**Figure 3.**
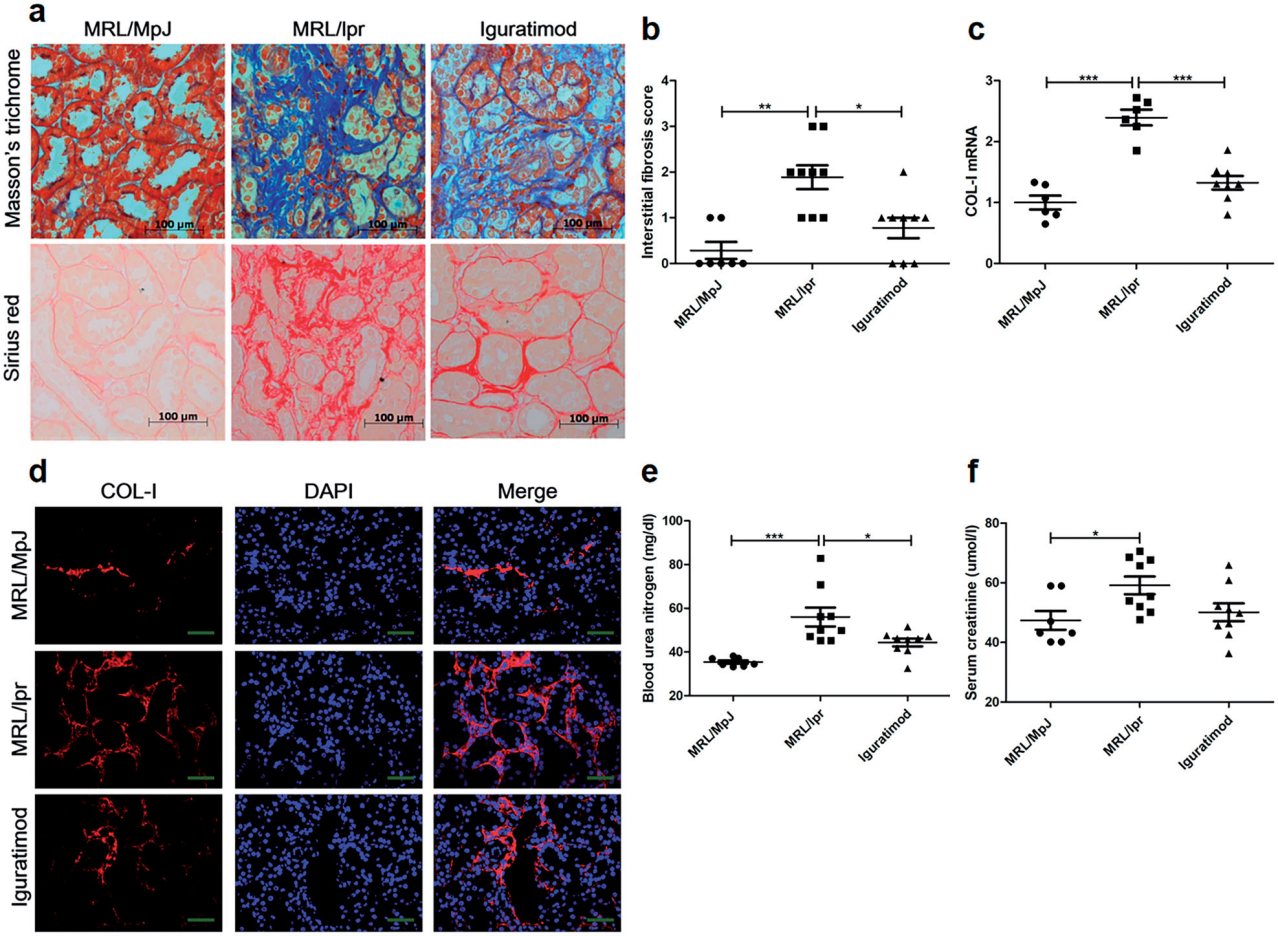
Iguratimod inhibited renal interstitial deposition of collagen fibers. (a) Collagen fiber deposition in renal interstitium determined by Masson trichrome and Sirius Red staining. (b) Interstitial fibrosis scores were evaluated blindly by one pathologist (*n* = 7, 9, 9, respectively.). Renal COL-I expression was detected by qRT-PCR (c) and immunofluorescence staining (d) (*n* = 6, 6, 8, respectively.). Levels of blood urea nitrogen (e) and serum creatinine (f) were also determined (*n* = 7, 9, 9, respectively). Results are shown as means ± SEM. *p < .05, **p < .01, ***p < .001.

CKD is characterized by the extensive accumulation and deposition of extracellular matrix, resulting in widespread tubulo-interstitial fibrosis, and tubulo-interstitial fibrosis is the final common pathway in CKD [18]. We determined the deposition of collagen fibers, which comprise the main component of extracellular matrix during tubulo-interstitial fibrosis, by staining cells with Masson trichrome and Sirius Red. We did not detect collagen fiber deposition in the renal interstitium of MRL/MpJ mice, but deposition was remarkable in MRL/lpr mice (Figure 3(a,b)). Remarkably, tubulo-interstitial fibrosis was significantly alleviated in iguratimod-treated MRL/lpr mice. The qRT-PCR findings revealed more COL-I in MRL/lpr than in MRL/MpJ mice, whereas iguratimod suppressed COL-I mRNA expression in MRL/lpr mice (Figure 3(c)). A comparison of COL-I deposition as immunofluorescence staining intensity showed obviously decreased COL-I accumulation in the renal interstitium of MRL/lpr mice administered with iguratimod compared with MRL/lpr mice without iguratimod administration (Figure 3(d)). Taken together, these findings indicated that iguratimod inhibited tubulo-interstitial fibrosis in mice with lupus.

Because deteriorating renal function reflects the substantial harmful effects of tubulo-interstitial fibrosis, we measured renal function indicators including blood urea nitrogen and serum creatinine. Levels of both were decreased in MRL/lpr mice administered with iguratimod compared with MRL/lpr mice without iguratimod administration (Figure 3(e,f)), although the differences in serum creatinine did not reach statistical significance.

### Iguratimod inhibited epithelial-to-mesenchymal transition in vivo

Myofibroblasts are the main producers of extracellular matrix during tubulo-interstitial fibrosis. Numerous myofibroblasts are derived from tubular epithelial-to-mesenchymal transition (EMT) [19,20], during which cuboidal renal tubular epithelial cells become elongated. This is accompanied by the decreased expression of epithelial cell markers such as E-cadherin, and the increased synthesis of mesenchymal cell markers such as fibronectin, α-smooth muscle actin, and FSP-1 [3]. Therefore, the expression of FSP-1 and E-cadherin in the kidney was investigated using immunofluorescence staining and qRT-PCR.

**Figure 4.**
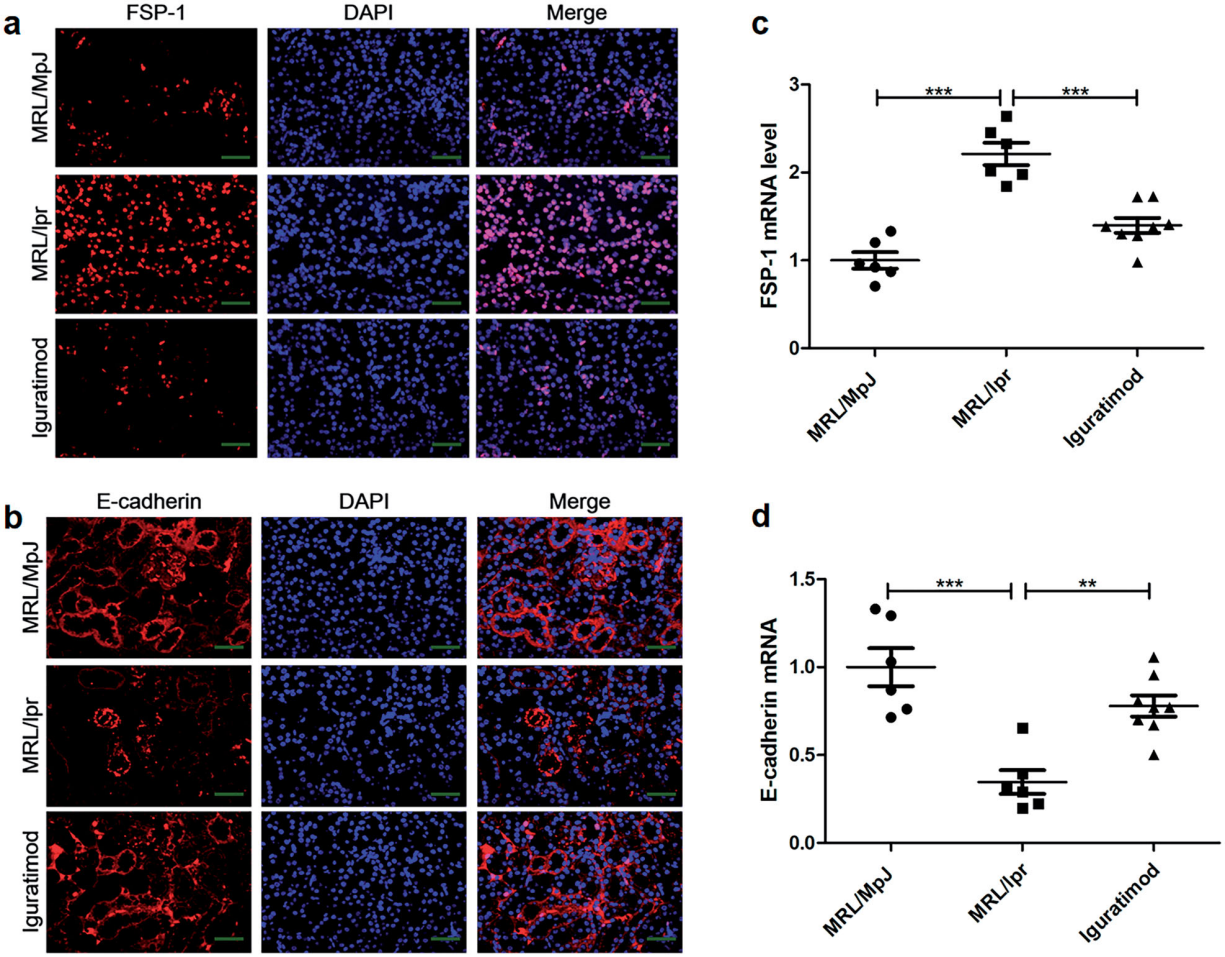
Iguratimod downregulated FSP-1 expression and upregulated E-cadherin expression in renal tubule-interstitium. Kidney tissues were stained with immunofluorescence to assess changes in levels of FSP-1 (a) and E-cadherin (b) protein among three groups. Renal FSP-1 (c) and E-cadherin (d) mRNA expression were detected by qRT-PCR. Scale bar, 100 μm. Data are shown as means ± SEM. *n* = 6, 6, 8, respectively. **p < .01, ***p < .001.

Figure 4(a) shows a few FSP-1(+) cells located in the renal interstitium of MRL/MpJ mice, but more tubular epithelial cells became positive for FSP-1 in MRL/lpr mice. Iguratimod inhibited FSP-1 expression in renal tubular epithelial cells (Figure 4(a)) and upregulated the tubular expression of E-cadherin, which is normally reduced in MRL/lpr mice (Figure 4(b)). Changes in the gene expression of FSP-1 and E-cadherin determined by qRT-PCR were consistent with the results of immunofluorescence staining (Figure 4(c,d)). These findings suggested that iguratimod inhibits tubular EMT in MRL/lpr mice.

### Iguratimod prevented TGF-β1-induced EMT in vitro

We further evaluated the role of iguratimod on tubular EMT *in vitro* to confirm the inhibitory effect of iguratimod on tubular EMT using human proximal tubular epithelial cells (HK2 cells) induced by TGF-β1, which is considered to be the most powerful fibrogenic inducer [21,22].

After incubation with 10 ng/mL TGF-β1 for 48 h, the HK2 cells became slender, and their growth became chaotic (Figure S1 in Supporting Information). However, iguratimod prevented these morphological changes and improved HK2 cell growth.

**Figure 5.**
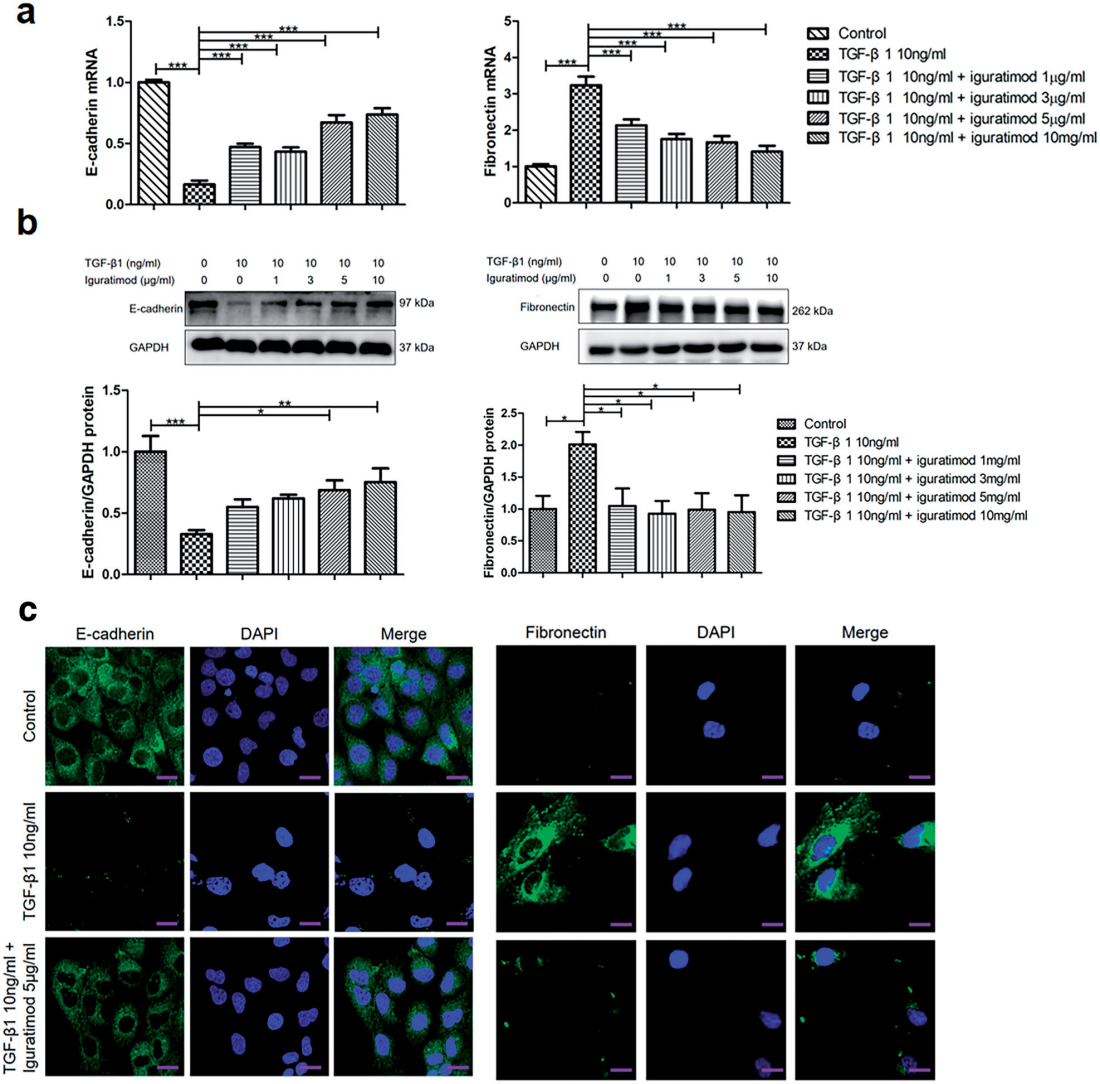
Iguratimod prevented TGF-β1-induced E-cadherin downregulation and fibronectin upregulation in HK2 cells. HK2 cells were incubated with 10 ng/mL TGF-β1 for 48 h, or with indicated concentrations of iguratimod for 2 h, followed by TGF-β1. Levels of E-cadherin and fibronectin mRNA and protein expression were detected by qRT-PCR (a), western blotting (b) and immunofluorescence staining (c), respectively. Cell nuclei were stained with DAPI. Scale bar, 200 μm. Results are shown as means ± SEM. *p < .05, **p < .01, *** *p* < .001.

We further analyzed the expression of E-cadherin and fibronectin in HK2 cells. Although TGF-β1 downregulated E-cadherin expression, the results of qRT-PCR and western blotting showed that iguratimod significantly increased E-cadherin expression (Figure 5(a,b)). In agreement with these results, immunofluorescent staining also showed that iguratimod prevented the TGF-β1-induced downregulation of E-cadherin (Figure 5(c)). Fibronectin was upregulated by TGF-β1 in HK2 cells, whereas iguratimod inhibited the increase in fibronectin expression at the gene and protein levels (Figure 5(a–c)).

### Iguratimod inhibited phosphorylation of TGFβRII, Smad2/3, and p38 MAPK and blocked nuclear translocation of β-catenin

**Figure 6.**
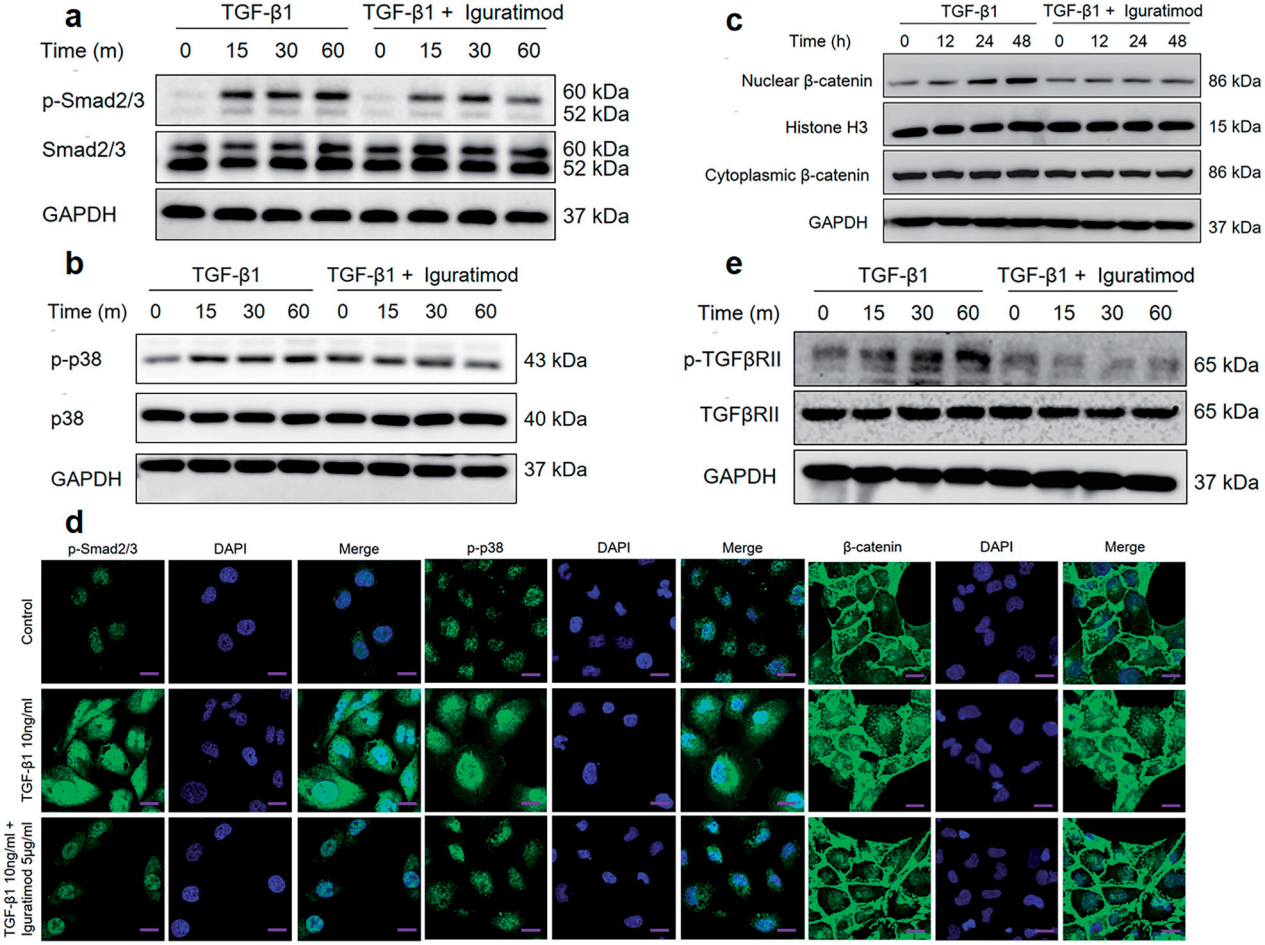
Iguratimod inhibited TGF-β1-induced activation of Smad and p38 MAPK signaling pathways. HK2 cells were incubated with 10 ng/mL TGF-β1 or with 10 ng/mL TGF-β1 and 5 μg/mL iguratimod for various amounts of time, then p-Smad2/3, Smad2/3 (a), p-p38 MAPK, p38 MAPK (b) nuclear β-catenin, cytoplasmic β-catenin (c), p-TGFβRII and TGFβRII (e) were detected by western blotting. (d) p-Smad2/3, p-p38 MAPK and β-catenin were analyzed by immunofluorescence staining, and cell nuclei were stained with DAPI. Scale bar, 200 μm.

The canonical Smad signaling pathway plays the most important role in TGF-β1-induced EMT [23], and the p38 MAPK signaling pathway also participates in the regulation of EMT induced by TGF-β1 [24,25]. In addition, TGF-β1 could activate Wnt/β-catenin signaling pathway and induce nuclear translocation of β-catenin, leading to EMT [26]. Therefore, we investigated whether iguratimod inhibits the activation of Smad, p38 MAPK and Wnt/β-catenin signaling pathways induced by TGF-β1. The western blotting results showed that iguratimod significantly blocked the phosphorylation of Smad2/3 and p38 MAPK and the nuclear translocation of β-catenin induced by TGF-β1 (Figure 6(a–c)). The immunofluorescent staining findings showed that iguratimod decreased the cytoplasmic and nuclear levels of phospho-Smad2/3 (p-Smad2/3) and p-p38 MAPK and nuclear levels of β-catenin increased by TGF-β1 (Figure 6(d)).

TGF-β1 first binds to TGFβRII, leading to the autophosphorylation of TGFβRII, thereby mediating downstream signal transmission [27], so p-TGFβRII is detected. Figure 6(e) showed that iguratimod inhibited the phosphorylation of TGFβRII induced by TGF-β1.

## Discussion

Iguratimod is a novel immunomodulatory agent that is widely applied by Chinese and Japanese rheumatologists to treat RA [28]. Here, we analyzed the effects of iguratimod on tubulo-interstitial injury in LN. Iguratimod inhibited tubulo-interstitial lesions *in vivo*, including immune deposition along the tubular basement membrane, infiltration of inflammatory cells, tubular injury, and fibrosis. *In vitro*, iguratimod reversed the TGF-β1-induced EMT process maybe through suppressing activation of the TGFβRII-Smad/p38 MAPK/β-catenin signaling pathways.

The tubulo-interstitium accounts for 90% of the total renal volume, and proximal renal tubular epithelial cells are the main cell types [14]. Although tubulo-interstitial damage is associated with poor long-term renal prognosis, the mechanisms resulting in tubulo-interstitial damage in LN have not received much attention [3]. The deposition of immune complexes in the kidney mediated by autoantibodies is the initiating factor of LN, in which anti-dsDNA plays a key role [14]. Glomerular and tubulo-interstitial expression of fibronectin is increased in mice and patients with active LN, which colocalizes with IgG deposition, suggesting a pathogenic link between autoantibody deposition and increased extracellular matrix accumulation in LN [29–31]. Immune deposition within the tubulo-interstitium is associated with increased cytokine production, immune cell infiltration, and tubular atrophy [15]. Anti-dsDNA antibodies bind to resident renal cells, including tubular epithelial cells, and can induce extracellular matrix synthesis, which promotes the progression of renal fibrosis [32]. The present study found that iguratimod inhibited IgG deposition along the tubular basement membrane in mice with lupus, which contributes to the prevention of subsequent tubulo-interstitial damage in LN.

Renal interstitial inflammatory cell infiltration is one of the pathological features of LN [33]. Activated inflammatory cells release numerous inflammatory factors and pro-fibrosis factors, which can transform resting extracellular matrix-producing cells into myofibroblasts, resulting in extracellular matrix accumulation that eventually leads to tubulo-interstitial fibrosis [34–37]. Moreover, inflammatory factors can lead to the abnormal proliferation and apoptosis of renal tubular epithelial cells [14], resulting in tubular atrophy, casts, and dilation. After tubular injury, the function of movement, migration, secretion, and transformation of renal tubular epithelial cells significantly increased, and the normal tubulo-interstitial structure was destroyed [17,38,39]. Numerous chemokines, inflammatory factors, pro-fibrosis factors, and matrix proteins are concomitantly produced [3], which further aggravates damage to renal tubular epithelial cells and tubulo-interstitial fibrosis. Here, iguratimod not only inhibited the renal interstitial infiltration of inflammatory cells, but also alleviated tubular injury in MRL/lpr mice. The number of Ki-67(+) cells and caspase-3(+) cells decreased in MRL/lpr mice administered with iguratimod, further confirming the protective role of iguratimod on renal tubules in LN.

Tubulo-interstitial fibrosis is a common pathological manifestation and the ultimate result of various types of CKD [18,40]. Tubulo-interstitial fibrosis leads to the irreversible deterioration of renal function, necessitating renal replacement therapy and increasing the risk of death in patients [40,41]. The present study found that iguratimod significantly alleviated the deposition of collagen fibers in the tubulo-interstitium in MRL/lpr mice. Immunofluorescence staining and qRT-PCR verified that iguratimod decreased COL-I expression, further supporting the notion that iguratimod plays a protective role. Moreover, iguratimod decreased blood urea nitrogen and serum creatinine values, although the latter did not reach statistical significance, which might be due to the small sample size. Therefore, iguratimod could be an effective drug for inhibiting the progression of tubulo-interstitial fibrosis in LN.

Tubular EMT is a key step in tubulo-interstitial fibrosis [40,42,43]. Although a few renal interstitial myofibroblasts originate from the bone marrow, more are derived from local tubular EMT during renal fibrogenesis [44]. Decreased expression of epithelial markers together with a concomitant increase in mesenchymal markers have been detected in renal biopsies from patients with LN and these are associated with renal impairment, interstitial leukocyte infiltration, and tubulo-interstitial fibrosis [17,39]. The present study showed that iguratimod decreased the expression of FSP-1 and increased that of E-cadherin in renal tubular epithelial cells of MRL/lpr mice. Moreover, iguratimod not only reversed morphological changes in HK2 cells induced by TGF-β1, but also prevented the downregulation of E-cadherin and upregulation of fibronectin. Therefore, iguratimod inhibited tubular EMT, which helps to the prevention of tubulo-interstitial fibrosis.

As we have known, many pro-fibrosis factors involved in EMT regulation; among them, TGF-β1 is considered to be the most important one [45–47]. After TGF- β1 stimulation, TGFβRII undergo autophosphorylation, then bind to TGF-βRI and phosphorylate TGFβRI to form TGFβRII-TGFβRI complexes, which then lead to the phosphorylation and activation of downstream signal proteins, such as Smad and p38 MAPK [25,26]. The present results showed that iguratimod not only inhibited TGFβRII phosphorylation induced by TGF-β1, but also negatively regulated activation of the Smad and p38 MAPK signaling pathway. β-catenin, a key regulator of the canonical Wnt/β-catenin pathway, has been linked to renal fibrosis [48]. Upon activation, β-catenin is increased and translocated from the cytoplasm to the nucleus to induce the expression of its downstream target genes. Studies have found that TGF-β1 could induces nuclear accumulation of β-catenin in tubular cells, and that β-catenin targeting of certain genes results in EMT [49,50]. Gong et al. found that miRNA-200a could inhibit TGF-β1-induced EMT by directly targeting β-catenin in HK2 cells [26]. The present study found that iguratimod blocked the nuclear translocation of β-catenin. Therefore, iguratimod inhibited the TGF-β1-induced tubular EMT process maybe through suppressing activation of TGFβRII-Smad/p38 MAPK/β-catenin signaling pathways.

In conclusion, iguratimod eased tubulo-interstitial lesions in LN, especially tubulo-interstitial fibrosis. *In vitro*, iguratimod suppressed TGF-β1-induced activation of TGFβRII-Smad/p38 MAPK/β-catenin signaling pathways, which play important roles in the process of tubulo-interstitial fibrosis. Therefore, iguratimod might have potential as a drug for inhibiting the progression of tubulo-interstitial fibrosis in LN.

## Supplementary Material

Supplemental Material
