# Supplementary material for: Iguratimod alleviates tubulo-interstitial injury in mice with lupus
Source: Ren Fail. 2022 Apr 6;44(1):636–47. doi: 10.1080/0886022X.2022.2058962 (PMC9004506; doi:10.1080/0886022X.2022.2058962)
Supplement: Supplemental Material [file IRNF_A_2058962_SM1285.pdf]

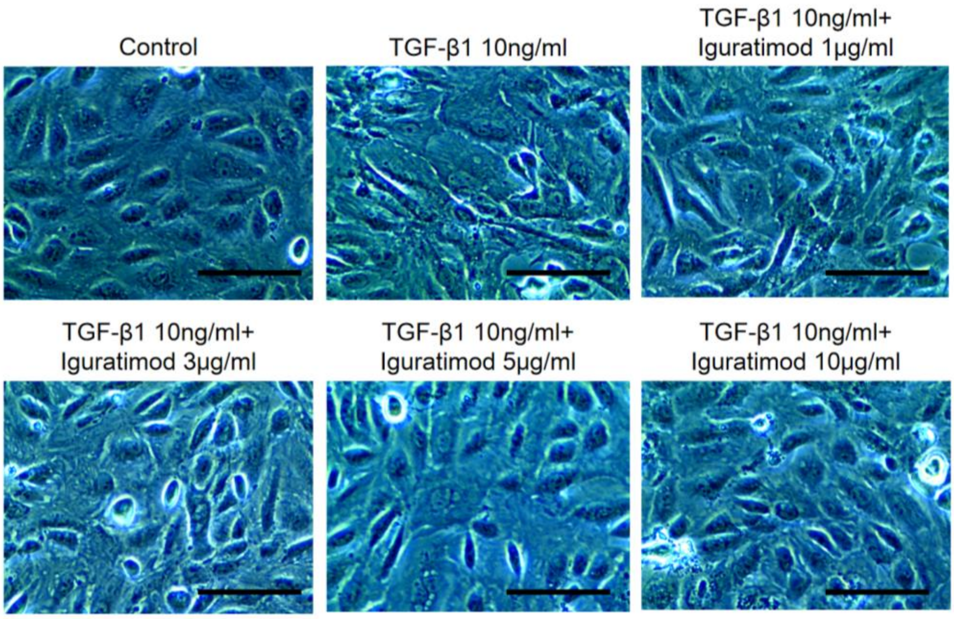

Fig. S1. Iguratimod prevented transforming growth factor β1 (TGF-β1)-induced epithelial-to-mesenchymal transition (EMT)-like morphologic changes in HK2 cells. For TGF-β1 alone treatment, human proximal tubular epithelial cells (HK2 cells) were treated with 10ng/ml TGF-β1 for 48 hours; for co-treatment with TGF-β1 and iguratimod, HK2 cells were preincubated for 2 h with iguratimod as indicated concentration, then incubated with TGF-β1. The morphology of HK2 cells was observed by microscope. After TGF-β1 treatment, the shape of HK2 cells became slender, and the growth of cells became chaotic, suggesting that EMT process is in progress; but co-treatment with iguratimod prevented TGF-β1-induced morphologic changes of HK2 cells in a concentration-dependent manner. Scale bar, 100 μm.
